# Viscosity-Sensitive Membrane Dyes as Tools To Estimate the Crystalline Structure of Lipid Bilayers

**DOI:** 10.1021/acs.analchem.3c01747

**Published:** 2023-08-01

**Authors:** Miguel Paez-Perez, Michael R. Dent, Nicholas J. Brooks, Marina K. Kuimova

**Affiliations:** MSRH, Department of Chemistry, Imperial College London, Wood Lane, London W12 0BZ, U.K.

## Abstract

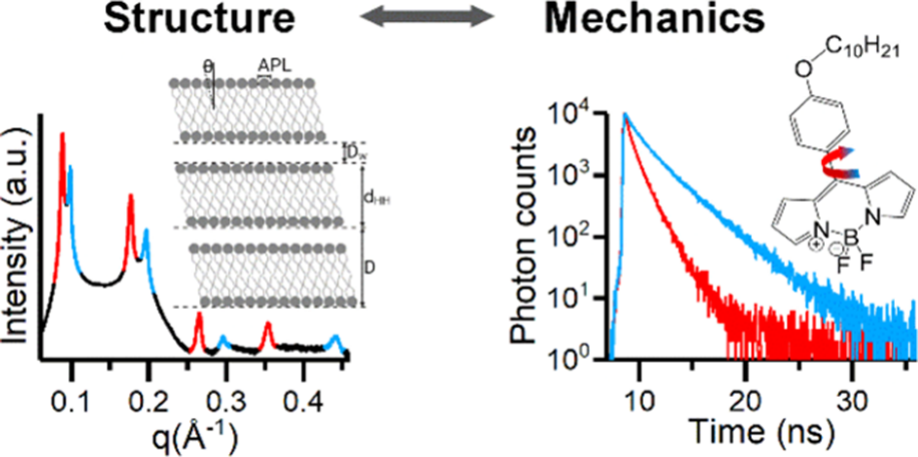

Lipid membranes are crucial for cellular integrity and regulation, and tight control of their structural and mechanical properties is vital to ensure that they function properly. Fluorescent probes sensitive to the membrane’s microenvironment are useful for investigating lipid membrane properties; however, there is currently a lack of quantitative correlation between the exact parameters of lipid organization and a readout from these dyes. Here, we investigate this relationship for “molecular rotors”, or microviscosity sensors, by simultaneously measuring their fluorescence lifetime to determine the membrane viscosity, while using X-ray diffraction to determine the membrane’s structural properties. Our results reveal a phase-dependent correlation between the membrane’s structural parameters and mechanical properties measured by a BODIPY-based molecular rotor, giving excellent predictive power for the structural descriptors of the lipid bilayer. We also demonstrate that differences in membrane thickness between different lipid phases are not a prerequisite for the formation of lipid microdomains and that this requirement can be disrupted by the presence of line-active molecules. Our results underpin the use of membrane-sensitive dyes as reporters of the structure of lipid membranes.

## Introduction

In addition to maintaining basic cellular integrity, lipid membranes are known to play important roles in cellular metabolism and are involved in cellular adaptation, homeostasis, and disease.^1^ This functionality arises from complex interactions between lipid molecules, which determines the membrane tension and viscosity. Under equilibrium conditions, the bilayer’s tension is minimized^2,3^ and, ultimately, this dictates the membrane’s mechanical properties, such as elasticity and viscosity; and structural parameters, including membrane thickness and lipid area.^4^ Importantly, minimization of the membrane tension may lead to lipid segregation into regions with distinct compositions and biophysical properties.^5,6^ These lipid microdomains are thought to play an important role in signal transduction and protein organization and therefore are of high biological interest.^7,8^

Many methods have been developed to study the structure of membranes. The high spatiotemporal resolution, capability of multiplexed labeling, and biocompatibility offered by fluorescence-based approaches have made such techniques a preferred method to study the lateral organization of biomembranes.^9^ Moreover, the use of environmentally sensitive dyes has enabled the study of the local molecular organization around the fluorescent probe at biologically-relevant timescales.^10,11^

The use of these fluorophores, including Laurdan,^12^ FlipTR^13,14^ or molecular rotors (MRs),^15^ has enabled the successful mapping of the membrane’s microenvironment.^12,13,16^ However, the photophysical properties of many of these molecules depend on multiple membrane parameters (e.g., microviscosity, polarity, or temperature) and, therefore, it is challenging to uniquely assign a physical descriptor to a given fluorescent readout. In fact, sometimes, a multiple parameter dependency prevents an accurate understanding of which biophysical property of a lipid bilayer is being measured by these sensors (e.g., whether they are sensitive to the lipid phase, membrane thickness, headgroup size, etc.)

**Figure 1:**
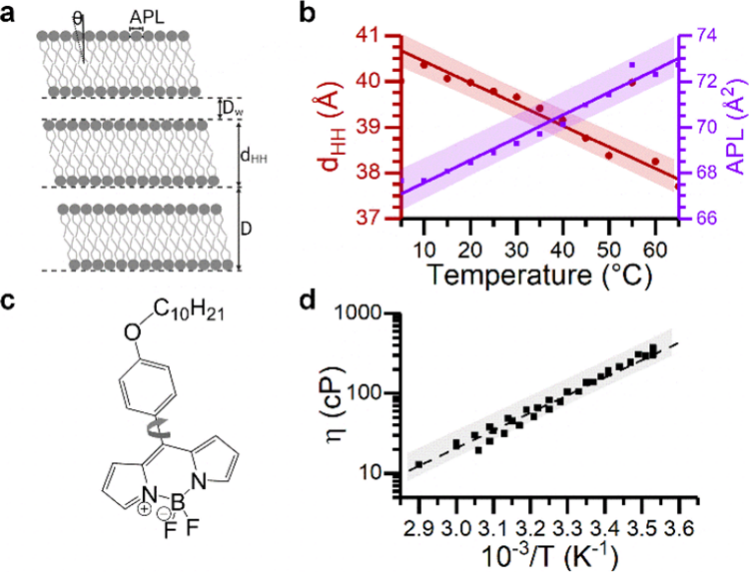
Effect of temperature on the lipid membrane structure and viscosity. (a) Structural parameters APL, tilt angle (Θ), *d*_HH_, water layer thickness (*D*_W_), and lamellar repeat spacing (*D*) are extracted from SAXS and WAXS diffraction patterns, see ESI. (b) Increasing the temperature leads to a higher degree of chain splay, which commonly results in a decrease of *d*_HH_ and an increase of the APL. (c) Structure of **BC10**. (d) Andrade’s relationship, η(*1*/*T*), between the membrane viscosity η and temperature *T* in DOPC membranes.

Uniquely, the fluorescence readout of BODIPY-based molecular rotors (Figure 1c) has been shown to be solely dependent on the local membrane microviscosity (η) within physiologically relevant values.^17,18^ This has enabled the quantitative measure of diffusion rates in lipid membranes^18,19^ and could, potentially be used to relate the bilayer mechanics to its molecular architecture. In molecular rotors, the nonradiative decay efficiency is coupled to the degree of intramolecular rotation. Hence, in less crowded, less viscous environments, nonradiative decay is preferred and therefore the MR’s fluorescence lifetime τ decreases, as predicted by the Förster–Hoffmann equation:^20^
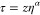
1where *z* and α are calibration constants that are experimentally determined by measuring the MR lifetime in solutions of known viscosity. We note that the molecular conformation of the BODIPY core (e.g., planar vs butterfly) has also been reported to affect the viscosity sensitivity of this fluorescent probe.^21^

Importantly, the fluorescence lifetime is independent of the local probe concentration and instrument setup, and this allows the direct quantitative mapping of microviscosity in heterogeneous model^19,22−25^ and cellular^25−30^ membranes under different stress conditions.^11,23,24,31^ Therefore, we anticipate the fluorescence lifetime of membrane-embedded BODIPY-based MRs could be used to directly infer changes in the membrane’s structure.

The structure and lateral organization of lipid membranes can be quantitatively probed using small and wide-angle X-ray scattering (SAXS/WAXS).^4^ SAXS diffraction patterns are used to elucidate the lipid mesophases and, in the case of lamellar structures, they report on the interlamellar distance, from which the membrane thickness (*d*_HH_) can be extracted. WAXS is sensitive to the in-plane membrane organization, and the position of the WAXS peak can be used to estimate the average area occupied by a lipid molecule (APL) within the membrane (Figure 1a).

When membranes contain different lamellar phases, distinct diffraction peaks appear in the SAXS regions, which arise from the difference in the domains’ thicknesses.^32−34^ However, phase separation could occur between domains with very similar thickness, as expected with highly dynamic nanosized membrane domains (sometimes described as lipid rafts),^35^ leading to the loss of multiple resolvable SAXS patterns. Alternatively, domains can also be distinguished by their different APL, which can cause the presence of multiple peaks in the WAXS range. Yet, liquid-disordered (L_d_) and liquid-ordered (L_o_) phases (which are thought to be related to membrane domain structures found in nature^35^) are characterized by similar distances between lipid molecules, and they are not easily distinguishable by WAXS.^36^

By using a combination of FLIM of the molecular rotor **BC10**, Figure 1c, and synchrotron SAXS/WAXS, we directly calibrate the fluorescence readout of this rotor against the structural parameters of model lipid membranes. We then explore whether such a relation holds true for other bilayer systems, including those displaying phase separation, where hydrophobic height mismatch drives the formation of domains with distinct viscosity. Finally, we challenge this relation by incorporating a line-active molecule, oleic acid, into phase-separated membranes and demonstrate how the presence of this lipid is able to disrupt the structural/mechanical relationship expected in canonical lipid bilayers.

## Experimental Methods

### Materials

Lipids 1,2-dioleoyl-sn-glycero-3-phosphocholine (DOPC), 1,2-dipalmitoyl-sn-glycero-3-phosphocholine (DPPC) and *E. coli* polar lipid extract were purchased from Avanti Polar Lipids dissolved in CHCl_3_ (25 mg/mL). Oleic acid (OA) and cholesterol (Chol) were obtained from Sigma Aldrich and dissolved in CHCl_3_ to a stock concentration of 50 mg/mL. Molecular rotor **BC10** was synthesized in house according to a previously published literature procedure (ref (28)). All other reagents were purchased from Sigma Aldrich, VWR, or Across Organic and used without further purification. Solvents for fluorescence studies were of spectrophotometric grade.

### Large Unilamellar Vesicle Formation

Large unilamellar vesicles (LUVs) were prepared by extrusion. Shortly, lipids in CHCl_3_ were mixed with either **BC10** or Laurdan at 0.5%mol and the organic solvent was evaporated off under a nitrogen stream. The resulting dry lipid film was further dried under vacuum for >2 h to remove any solvent traces. Subsequently, the film was hydrated with water to a final lipid concentration of 1 mM and vortexed to yield a cloudy solution of polydisperse multilamellar vesicles. This mixture was then extruded above the lipid’s melting transition temperature through a 200 nm polycarbonate filter to yield a monodisperse LUV population (average diameter of ∼180 nm determined by DLS).

### Spectroscopic Characterization of LUVs

LUVs were diluted 10-fold and placed into quartz cuvettes (10 mm path length). Emission spectra of Laurdan-labeled vesicles were acquired using a Horiba Yvon Fluormax 4 fluorimeter after 360 nm excitation, from which the Laurdan’s general polarization (GP) was calculated as:
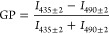
2

Time-resolved fluorescence decay traces of **BC10**-labeled liposomes were acquired using a Horiba Jobin Yvon IBH 5000 F time-correlated single photon counting (TCSPC) instrument. A pulsed 404 nm diode (NanoLED) was used to excite **BC10**, and fluorescence was detected at 515 nm. The acquisition was stopped after peak counts reached 10.000, and the resulting traces were fitted using DAS software to the minimum number of decay components (2 for gel-phase membranes, 1 for liquid-phase bilayers), ensuring the fitting metric χ^2^ < 2. The longer lifetime component was used as a viscosity descriptor for the gel phase membranes, as described in ref (19).

The measured lifetime was then used to estimate membrane viscosity based on the Förster–Hoffmann equation with the parameters given by Hosny et al.:^37^
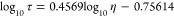
3

The temperature was controlled by a Peltier cell (fluorimeter, error: ±0.5 °C) or a water bath (TCPSC, error: ±1 °C) and was left to equilibrate for at least 5 min before each measurement.

### X-ray Diffraction Experiments

Dry samples of a given lipid mixture (20 mg total mass) were hydrated with DI water to 70% w/w and subjected to 15 freeze-thaw cycles to ensure uniform mixing. Samples were then loaded into 2 mm diameter polymer capillary tubes and sealed. SAXS and WAXS measurements were performed at beamline I22, Diamond Light Source, UK.^38^ Experimental uncertainties for OA experiments were estimated from duplicate, independent measurements.

### Giant Unilamellar Vesicle (GUV) Formation

Around 30 μL of 1 mg/mL (total lipid, at the desired DOPC:OA:DPPC:Chol ratio and supplemented with 0.5%mol **BC10** or Laurdan) was spread onto an ITO slide to create a thin lipid film. CHCl_3_ traces were removed by drying overnight in a desiccator. A polydimethyl siloxane (PDMS) spacer with a thickness of ∼2 mm was then placed on top of the ITO slide to create a chamber, which was then filled with a 0.4 M sucrose solution and then sealed using a second ITO slide. GUVs were electroformed (at 60 °C) by applying an electric field of 1 *V*_pp_@10 Hz for 90′ followed by a detachment phase of 1 *V*_pp_@2 Hz for 30′. Finally, giant unilamellar vesicles (GUVs) were gently recovered by tilting the chamber (avoiding pipetting in the process).

### Confocal Laser Scanning Microscopy (CLSM)

Microscopy images were obtained on a Leica SP5 II inverted confocal microscope using a 20× (NA:0.7) dry objective. A Ti:Sapphire laser (Coherent, Chameleon Vision II, 80 MHz) provided two-photon excitation (900 nm), and fluorescence emission was collected either between 425–465 and 480–520 nm for Laurdan. Laurdan’s GP function was calculated as:
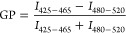
4

### Fluorescence Lifetime Imaging Microscopy (FLIM)

FLIM micrographs were obtained on a Leica SP5 II inverted confocal microscope using a 20× (NA:0.7) dry objective. The Ti:Sapphire laser (Coherent, Chameleon Vision II, 80 MHz) provided two-photon excitation (at 930 nm), and **BC10** fluorescence emission was collected between 500 and 580 nm. FLIM images were acquired using a TCSPC card (Becker & Hickl GmbH, SPC-830). Instrumental response function (IRF) was obtained using the second harmonic generation signal from urea crystals. Pixel-wise fitting of the fluorescence decays (Figure S12) was done by fitting the decays to a monoexponential model (minimum of 200 counts/pixel after binning) using the commercially available software SPCImage. **BC10** lifetimes obtained with SPCImage or a custom-written script (see ESI) were transformed to viscosity values according to eq 3. The similarity between **BC10** lifetime in LUVs and GUVs was taken as an indicator of the anticipated lipid:dye ratio in both cases and the lack of significant lipid oxidation in GUVs.

### Statistical Analysis and Data Representation

Scatter plots display the mean ± S.D. Box plots display the 25–75% range, error bars represent ±S.D., and median is shown by a horizontal line and mean by a dot. Origin software was used to perform a one-way ANOVA test. *p < 0.05; **p < 0.01; ***p < 0.001. For the temperature scans, solid lines represent the values obtained by linear fitting of the experimental data (dots) presented in the graph. The shadowed area corresponds to the 95% CI of the linear fit.

## Results and Discussion

### Membrane Microviscosity Correlates with the Structural Parameters of the Lipid Bilayer

Initially, we explored the relationship between the bilayer’s structure (*d*_HH_ and APL), measured by SAXS/WAXS, and its microviscosity, measured by the molecular rotor **BC10**, by systematically increasing the temperature. We previously demonstrated that the **BC10** response is temperature independent, i.e., the lifetime measured is only affected by the viscosity, independent of the measurement temperature.^18^

**Figure 2:**
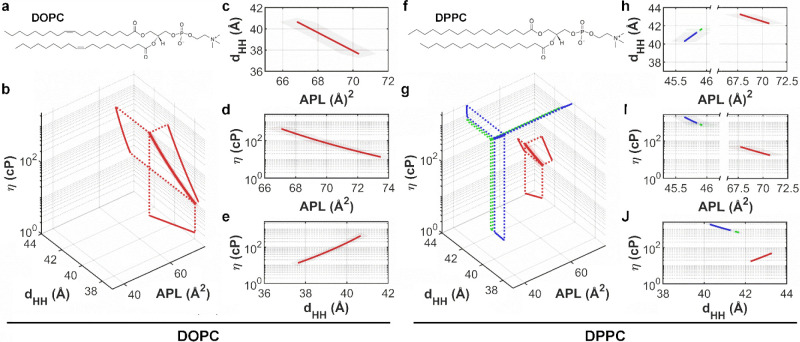
Relationship between the membrane’s structural and mechanical properties as a function of the lipid phase. (A, F) Molecular structures of DOPC and DPPC. (B, G) 3D plots showing the interrelations between *d*_HH_, APL, and η, as measured by **BC10**. Corresponding 2D projections of the 3D plots: (C, H) *d*_HH_ vs APL plots. (D, I) Relationship between η and APL. (E, J) Influence of *d*_HH_ on η. Color coding corresponds to tilted gel L_β’_ (blue), ripple P_β’_ (green), and fluid L_α_ (red) lipid phase.

The gain in thermal energy leads to an increased motion of the lipid’s alkyl chains, this would increase the area per lipid, and decrease both the membrane thickness and microviscosity. Measurements performed on 1,2-dioleoyl-sn-glycero-3-phosphocholine (DOPC) bilayers confirmed this trend (Figures 1b, 2b–e, and S1–3). This lipid remains in the fluid lamellar phase (L_α_) throughout the chosen temperature range owing to its two unsaturated chains, and its behavior has been studied extensively; hence, we selected DOPC as our standard sample. We observed that heating DOPC membranes from 5 to 65 °C caused a gradual decrease in membrane thickness, *d*_HH_, from 40.7 ± 0.2 to 37.6 ± 0.2 Å and an increase in APL from 67.1 ± 0.5 to 73.5 ± 0.5 Å^2^ (Figures 2b–e and S1,S2). These changes are linear with a slope of (−4 ± 0.2) × 10^–2^ Å/°C and (10 ± 0.5) × 10^–2^ Å^2^/°C, respectively, consistent with previous results for fluid membranes.^39,40^ These variations were accompanied by a decrease in the membrane microviscosity (Figures S2 and S3a) from ∼420 to ∼10 cP. **BC10** time-resolved decays conform to a monoexponential function, consistent with a single dye environment in the L_α_ phase. Notably, the change in DOPC viscosity with temperature followed the log-inverse relation described by Andrade’s model (eq 5, Figure 1d), which suggests that the DOPC bilayer behaves analogously to an ideal liquid.
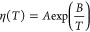
5

We also observed a negative correlation between *d*_HH_ and APL (Figure 2c), corresponding to a positive Poisson ratio (ν) typical of bilayers in the fluid phase, where they become thinner as they stretch, in agreement with previous simulations^41^ and experiments.^42^

Next, we tested Laurdan (Figure S3d) to investigate whether this commercially available probe displayed similar sensitivity to changes in the membrane structure. In this case, the environmental sensitivity of Laurdan comes from the presence of a dipole moment along its naphthalene moiety, which ultimately leads to a reorientation of the solvent molecules around the dye and a red shift of the emission spectra in polar/hydrated environments.^43^ Therefore, changes in lipid order and membrane hydration will cause a spectral shift for Laurdan’s fluorescence.^12^ Such changes are commonly quantified using the GP function (eq 2).^44,45^ Compared to **BC10**, Laurdan locates closer to the membrane-water interface;^46^ hence, its readout will be influenced differently compared to that of the MR. Increasing temperature resulted in a red shift of Laurdan’s fluorescence maximum in DOPC membranes (Figure S3c). However, there was a lack of linear relationship at increasing temperature, between the response of Laurdan’s GP and membrane viscosity reported by **BC10** (Figure S4a), suggesting that these dyes sense different membrane properties. We conclude based on the lack of linearity (Figures S2 and S4a) that the response of Laurdan cannot be directly related to the bilayer’s structure; and this prevents its use as a tool for direct quantification of the membrane’s properties.

Next, we investigated whether **B10** fluorescence lifetime still shows a correlation with structural parameters measured by SAXS/WAXS in bilayers displaying a more complex phase behavior, such as DPPC. The fully saturated DPPC lipids experience stronger intermolecular attractive forces, and this significantly decreases DPPC in-plane motion, causing the membrane to be arranged in a highly ordered tilted gel (L_β’_) phase. As the temperature is increased, the attractive interactions become weaker and the gel membrane transitions towards a ripple (P_β’_) phase and, finally, to the L_α_ phase, analogous to DOPC membranes, at temperatures exceeding 41 °C.

At room temperature, the DPPC L_β’_ phase is evidenced by the two peaks observed in the WAXS pattern (Figure S5b). Upon heating, an increase of both *d*_HH_ and APL was observed (Figure 2g,h), suggesting a negative Poisson ratio, ν = (−9 ± 0.1) × 10^–2^. This behavior can be attributed to a decrease of the lipid tilt, which outweighs the reduction in length of the hydrocarbon chains due to the increase in chain motion, and to a small change in chain length, effectively fully extended in gel phase lipids (Figure S2).^47^ Altogether, this results in both higher membrane thickness and APL with increased temperature. In **BC10** lifetime measurements, we also detect a biexponential decay for the DPPC L_β’_ phase, which was previously assigned to two possible localizations of the rotor, relative to the lipid tails (Figure S3b). The longer component is characteristic of the lipid bilayer viscosity^19^ and calculated viscosity shows three distinct regions of linearity vs 1/*T*, according to the Andrade equation, with a separate slope for each of the phases: L_β’_, P_β’_ and L_α_ (Figure S2). Overall, the L_α_ phase of DPPC displays very similar behavior to DOPC for all parameter interdependencies (Figure 2, red). However, different behaviors are seen for the gel phase (Figure 2, blue); e.g., the higher slope of the function of the membrane viscosity vs APL is observed in the gel (Figure 2i,j, blue), compared to the L_α_ phase (Figure 2i,j, red). This is consistent with a lack of stress buffering capacity provided by the greater structural flexibility of fluid membranes. Also, a negative slope for viscosity vs *d*_HH_ is observed in the gel phase (Figure 2j, blue). These data suggest that the correlation between the structural parameters of the lipid bilayer and viscosity is strongly phase-dependent.

### Structure–Viscosity Relationship Is Maintained for Membranes in the Same Phase Regardless of Lipid Composition

After measuring the relationship between the structure and microviscosity of DOPC and DPPC membranes, we explored whether the observed trends could be exploited to infer the structural properties of lipid bilayers with a different or unknown composition. Given the linear relationship between the temperature and the XRD-derived parameter  (either *d*_HH_ or APL) of the form:
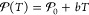
6where  is the structural parameter at 0 K and *b* is the thermal coefficient; it is possible to combine eqs 5 and 6:
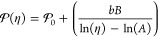
7

We focused on the APL as a structural descriptor, as we have found it has a greater dependency on viscosity, compared to *d*_HH_, for DOPC. Subsequently, we used the lifetime of **BC10** to measure the microviscosity of 1-palmitoyl-2-oleoyl-glycero-3-phosphocholine (POPC) and 1,2-dilauroyl-sn-glycero-3-phosphocholine (DLPC) fluid membranes (containing one and no points of unsaturation, respectively), and mapped those values onto the corresponding APL obtained from SAXS/WAXS measurements (Figure S6), according to eq 7.

**Figure 3:**
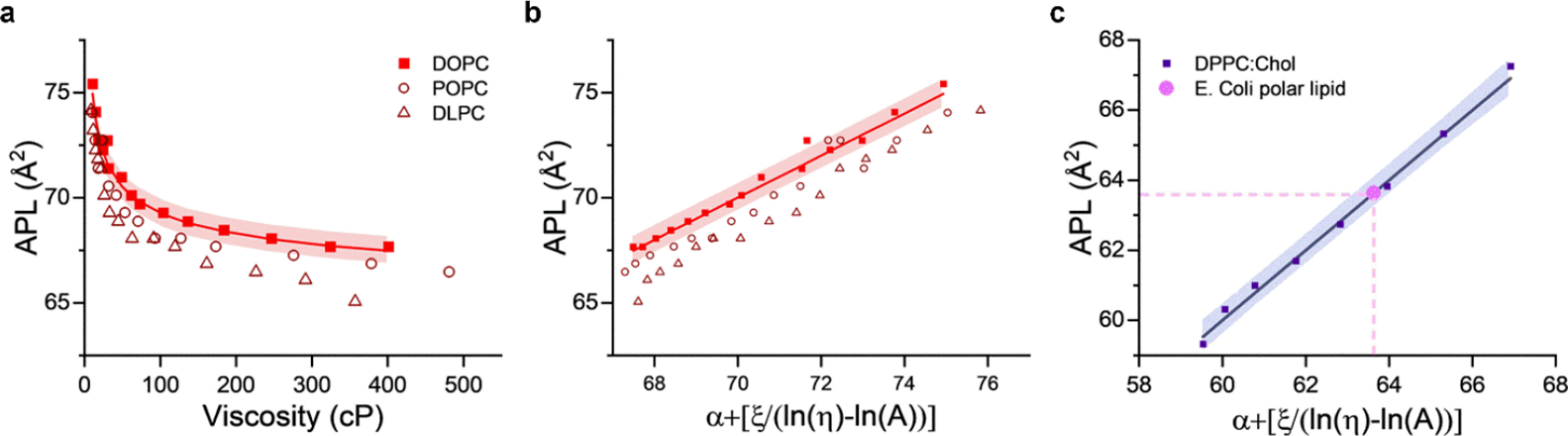
Membrane’s viscosity–structure relationship is maintained in fluid lipid bilayers. (A) Relationship between APL and microviscosity, reported by **BC10**. (B) Relationship between APL and the transformed viscosity according to eq 7, showing a small lipid-dependent offset for various lipids. (C) Determination of APL in *E. coli* membrane following structure-viscosity calibration in synthetic membranes with similar biophysical properties.

As seen in Figure 3b, the temperature response (i.e., the slope) of all fluid phase membranes was almost identical, with a small offset between the datasets for various lipids, which could be anticipated from the  term in eq 7. However, if a numerical value for this term is obtained either through empirical relations (Figure S8) or *in-silico* methods, and the membrane composition is not significantly altered during the experiment, eq 7 could be used to derive changes in the membrane’s structural parameters, by using molecular rotor’s fluorescent readout. We note that this relationship did not hold for bilayers in the gel phase (Figure S7).

We then sought to infer the APL of biologically relevant, *E. coli-*derived polar lipid extracts (ECPLE), using the calibration obtained from a synthetic lipid mixture. **BC10** measurements in ECPLE LUVs gave the membrane viscosity of ∼245 cP at 25 °C (Figure S9), comparable to our previous reports.^48^ Since *E. coli* membranes have been reported to display behavior similar to the liquid-ordered (L_o_) phase,^48,49^ we performed the structure-viscosity calibrations using 70:30 DPPC:Cholesterol (Chol) liposomes, which are known to be in the L_o_ phase.^50^ Given the closeness of viscosity values (∼308 cP at 25 °C), it could be considered a good match. As depicted in Figure 3c, mapping ECPLE viscosity onto the APL of DPPC:Chol calibration curve suggested a mean APL_ECPLE_ ∼ 63.6 Å^2^, in good agreement to the mean APL directly obtained from WAXS measurements, APL_ECPLE_ ∼ 64.3 Å^2^ (Figure S10).

Overall, these results show that for a known response of an environmentally sensitive membrane probe, such as **BC10**, it should be possible to relate the fluorescent readout to the molecular architecture of lipid membranes, in a certain phase. Such relation has the potential of being useful to infer alterations in the membrane’s structure that are not measurable with XRD, e.g., in lipid bilayers *in-cellulo*.

### Combined XRD/FLIM Characterization of Ternary Lipid Mixtures Reveals the Relationship between Structure and Viscosity in Phase-Separated Membranes

Next, we increased the complexity of our model membranes by combining DOPC, DPPC, and cholesterol (Chol) lipids. This mixture is known to exhibit microscopic phase separation between DOPC-rich liquid-disordered (L_d_) domains and DPPC/Chol-rich liquid-ordered (L_o_) domains,^51^ and has been used as a model system of the membrane heterogeneity suspected to occur in cellular membranes, the so-called “lipid rafts”.

**Figure 4:**
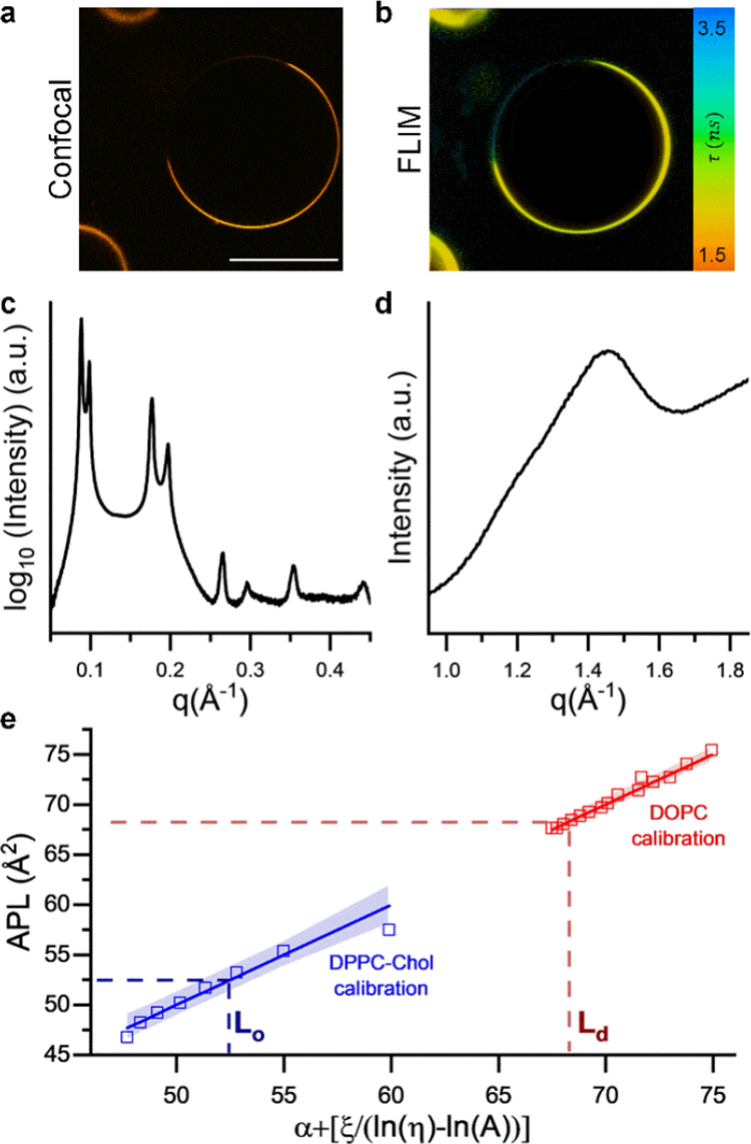
**BC10** can be used to infer the structural properties of membrane domains. (A) Confocal and (B) FLIM images of 40:40:40 DOPC:DPPC:Chol GUVs. Scalebar: 30 μm. (C) SAXS and (D) WAXS traces corresponding to the lipid mixture used in (A). While bilayer thickness can be easily extracted from the clearly defined SAXS peaks, estimation of the APL from the WAXS pattern is challenging. (E) Calibration of APL vs viscosity (eq 7) in known lipid mixtures allows to estimate the APL in the L_d_ and L_o_ phases. Phospholipid APL for the L_o_ phase has been corrected for cholesterol contribution (see ESI for details).

We electroformed 40:40:20 (%mol) DOPC:DPPC:Chol GUVs and measured the lifetime of **BC10** within the membrane using fluorescence lifetime imaging microscopy (FLIM). Figure 4b reveals two distinct regions (L_d_ and L_o_) with viscosities of 197 ± 33 and 413 ± 128 cP, respectively, in agreement with previous reports.^19^ L_d_/L_o_ phase separation was further confirmed by two clearly defined peaks in the SAXS region (Figure 4c) which correspond to membrane thicknesses of 40.5 ± 0.6 and 46.1 ± 0.2 Å for the L_d_ and L_o_ phases, respectively. These distinct values for the two phases are consistent with those previously reported^50,52,53^ and exemplify how hydrophobic mismatch is a driving force for lipid phase separation. In addition, these (η, *d*_HH_) data pairs show a reasonable correlation with those observed in pure DOPC membranes (39.9 Å, 166 cP at 20 °C) and DPPC bilayers (41.28 Å, 510 cP at 30 °C). The higher bilayer thickness of DPPC-rich L_o_ regions can be attributed to the presence of cholesterol, which disrupts the lipid tilt.^54^

Although the presence of two phases was evident in the SAXS region, they could not be clearly resolved from the WAXS diffraction pattern (Figure 4d), thus preventing an accurate estimation of the lipid-lipid distance. Previous work suggested a 40:40:20 DOPC:DPPC:Chol membrane will contain a relative fraction of cholesterol in the L_d_ and L_o_ phases of ∼0.1 and 0.3, respectively.^55^

We decided to utilize our new correlation technique and to measure the lifetimes of **BC10** in different phases observed in GUVs, in order to map these values onto calibrations performed in pure DOPC and DPPC/Chol (70/30%mol) model membranes, Figure 4e, to obtain an estimate of the APL in the L_o_ and L_d_ phases. Our results (Figure 4e) suggested an area per phospholipid of 68.3 and 52.4 Å^2^ for the L_d_ and L_o_ phases, respectively, in good agreement with previous reports (∼67 and 52 Å^2^ for L_d_ and L_o_ regions).^50,55,56^ Overall, these results highlight the combination of viscosity-sensitive molecular rotor **BC10** and FLIM has the potential to be a proxy reporter of the membrane structure, including during phase separation.

### Line-Active Molecules Disrupt the Structural–Mechanical Relationship of Canonical Membranes

Traditionally, height mismatch (seen as separate peaks in SAXS) is considered as a driving force for phase separation in lipid membranes. We set out to test whether our approach could be applied when the hydrophobic height mismatch between the two lipid domains was minimal/not observable with SAXS. This condition has been postulated to be responsible for the transient nature of cellular “lipid rafts”,^35,57^ and we aimed to mimic it by incorporating line-active molecules, which accumulate at the domain boundary, reducing the line tension and the difference in membrane thickness.^58^

An example of one such molecule is OA. Evidence from epidemiological studies suggests that a higher proportion of monounsaturated fatty acids, such as OA, in the diet is linked with a reduction in the risk of coronary heart disease, which is possibly achieved through modification of lipid membrane composition.^59^ OA is known to increase the membrane curvature, thickness, and bending rigidity,^60^ while the effect on membrane order remains controversial.^61,62^ In addition, OA acts as a lineactant,^63^ reducing the line tension between L_d_ and L_o_ domains, and is thus able to modulate the lateral organization of phase-separated membranes, such as biologically relevant “lipid rafts”.^59,63^

Initially, we investigated the effect of OA in pure DOPC membranes, as both molecules are structurally related, as they both contain a cis-monounsaturated hydrocarbon chain. The addition of OA to DOPC resulted in a shift of the SAXS spectra to lower *q*-values, Figure S11a,b, indicative of thicker membranes—from 39.3 ± 0.1 to 46.1 ± 0.2 Å—up to 40%mol OA.^61^ Beyond 40%mol the high curvature imposed by the fatty acid led to the appearance of an inverted hexagonal (H_II_) phase, as described previously^60,64,65^ However, changes in the in-plane membrane distribution were minimal, as judged from the WAXS traces (Figure S11c,d). On the contrary, the addition of OA leads to an increase of the membrane’s bending rigidity^60^ and order.^61,66,67^ These measurements are consistent with our **BC10** data indicating an increase in membrane microviscosity from 185 ± 12 to 222 ± 25 cP after 40% DOPC was replaced with OA (Figure S12). This change in viscosity corresponded to a decrease in the average APL of −0.24 Å^2^ using the calibration depicted in Figure 3b, which was within the same order of magnitude as the one measured directly from the WAXS traces, −0.41 Å^2^.

**Figure 5:**
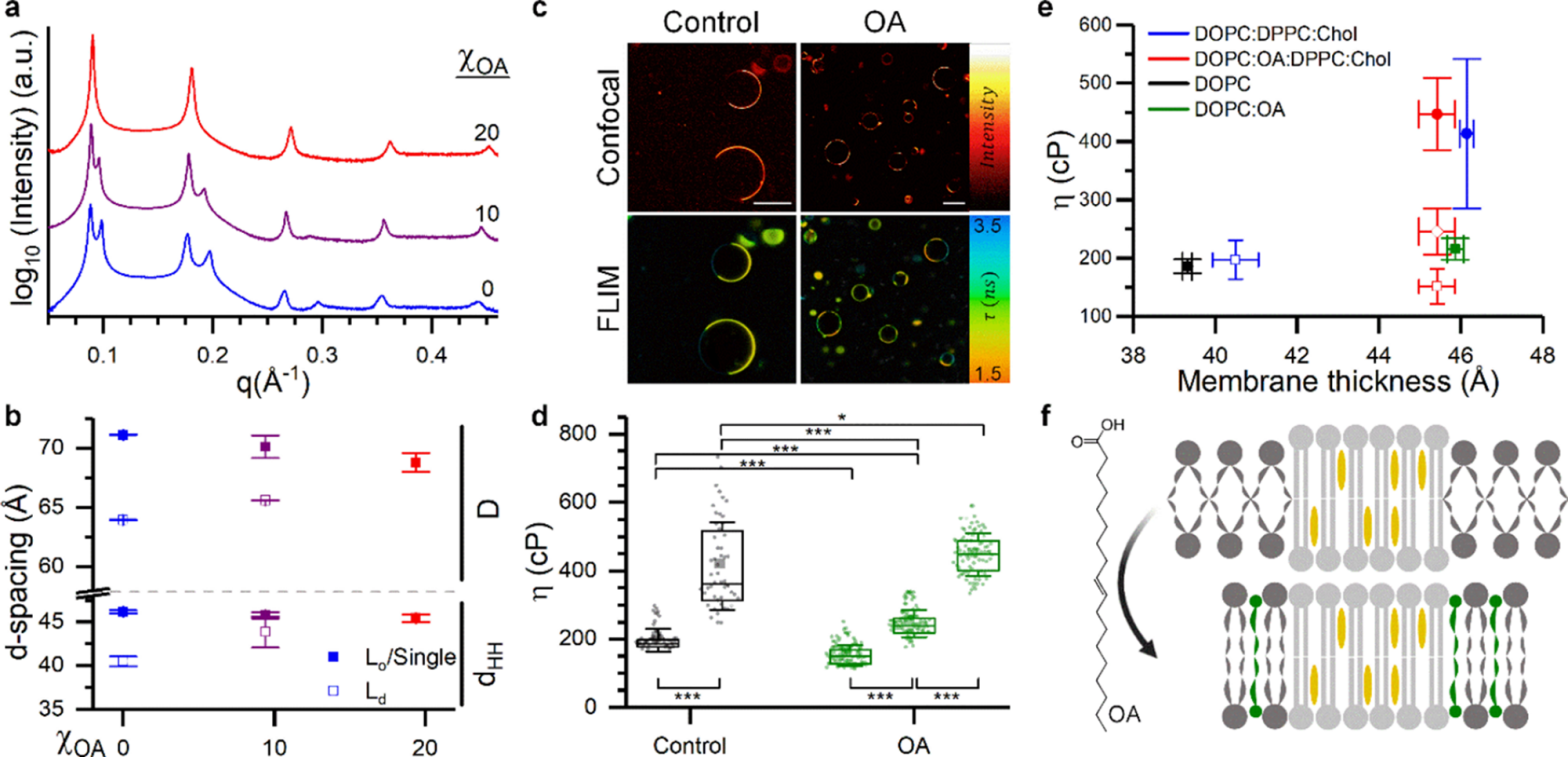
Effect of oleic acid (OA) on phase separation. (A) SAXS pattern from DOPC:OA:DPPC:Chol membranes with increasing %OA replacing DOPC. (B) Effect of increasing OA on lamellar repeat spacing and membrane thickness. (C) Confocal and FLIM micrographs of 40:0:40:40 and 20:20:40:20 DOPC:OA:DPPC:Chol GUVs. Scalebar: 30 μm. (D) Box plot showing the change in membrane viscosity upon OA addition. (E) Plot of membrane viscosity against membrane thickness. (F) Schematic showing a proposed interaction of OA with phase-separated membranes.

Next, we replaced DOPC with OA in phase-separated membranes, knowing that OA has a high affinity toward the L_d_ phase,^63^ to investigate whether the disruptive effect of this fatty acid was also observed in ternary lipid mixtures. As a result, we observed an increase in the lattice parameter and membrane thickness of the L_d_ phase, which saturated at *d*_HH_ ∼ 45.4 Å for OA concentrations above 20%, coupled with a slight decrease in the thickness of the L_o_ domains (Figures 5b and S13a,b). At this point, the signals from the L_d_ and L_o_ phases appeared to merge in the SAXS pattern, as evidenced by the presence of a single peak at 20% OA. Yet, our microscopy images clearly indicated the presence of distinct lipid domains (Figure 3c,d), in agreement with previous work by Shimokawa et al.^63^ thus suggesting optical probes allow to detect membrane domains not distinguishable through SAXS measurements.

Using FLIM measurements of **BC10**, it was clear that the membrane’s lateral organization was altered when DOPC was replaced with OA. In particular, adding OA at a 20% molar concentration to replace DOPC led to a change in the GUV morphology, where the total area corresponding to disordered regions decreased and appeared as multiple circular domains within a more ordered matrix (Figure S14). The lower degree of domain coalescence was likely a consequence of reduced line tension at the domain’s boundary, in agreement with the lower height mismatch between domains upon the addition of OA.^63^ In addition, our quantitative analysis (see ESI for details) of lifetime clusters in FLIM images (Figures 3d and S15) revealed the presence of three distinct regions of different viscosity (∼155 ± 30, ∼250 ± 45, ∼465 ± 75 cP), which could also be distinguished using a polarity sensitive dye Laurdan^12^ (Figure S16). We note that, while Laurdan shows a more homogeneous partitioning between the L_d_ and L_o_ phases compared to **BC10**, the use of self-calibrating readouts (GP and lifetime, respectively) will avoid any concentration-dependent artifacts of the probe localization in phase-separated membranes. We also estimated the APL for these regions from the **BC10** readings (as described in the previous section), obtaining approximate values of 68.6, 67.9, and 51.9 Å^2^, respectively. We discuss our hypotheses on the domain composition in the ESI.

Overall, these results suggest that the microscopic lipid phase separation is possible despite the absence of a significant height mismatch between the different domains, contrary to the common hypothesis.^68^ This situation was previously described by Mills et al., who proposed to use peak splitting in the SAXS region as a sufficient, but not necessary, condition for phase coexistence.^69^ Apart from the case described above, such occurrence could arise, for example, if metastable, antiregistered domains of different thicknesses are present.^70,71^

## Conclusions

The combination of small- and wide-angle X-ray scattering and FLIM imaging of molecular rotor **BC10** described here has enabled us to perform a combined structural and micromechanical characterization of lipid membranes exhibiting different lipid phases and types of lateral organization. We demonstrate how the calibration of the fluorescence readout of a molecular probe against known structural descriptors of the membrane allows the use of fluorescent dyes to derive quantitative information regarding the molecular organization of the lipid bilayer (e.g., the area per lipid), for both single-component membranes and bilayers containing multiple domains. Finally, we exploited this strategy to demonstrate how addition of a biologically relevant lineactant molecule, OA, led to lipid phase separation occurring without the hydrophobic mismatch, the most widely agreed driving force for domain formation. Such lipid arrangements may be of importance in biology, where the lack of hydrophobic mismatch can facilitate the formation of transient lipid nanodomains with distinct mechanical properties (“lipid rafts”) while still capable of undergoing easy lipid exchange. Overall, our approach expands the capabilities of environmentally sensitive membrane dyes, allowing the direct estimation of the membrane’s structural properties in physiologically relevant settings. Hence, our approach has the potential to help bridge the gap in the understanding of lateral structuring between model and biological membranes.

## Supporting Information Available

Experimental section including analysis of SAXS/WAXS patterns and FLIM images. Raw SAXS/WAXS spectra, effect of temperature on membrane descriptors, raw BC10 fluorescent decay traces, raw Laurdan emission spectra, effect of OA on DOPC membranes, Laurdan GP images in DOPC:OA:DPPC:Chol GUVs (PDF)
