# Supplementary material for: Viscosity-Sensitive Membrane Dyes as Tools To Estimate the Crystalline Structure of Lipid Bilayers
Source: Anal Chem. 2023 Aug 1;95(32):12006–14. doi: 10.1021/acs.analchem.3c01747 (PMC10433245; doi:10.1021/acs.analchem.3c01747)
Supplement: Supplementary file 1 — ac3c01747_si_001.pdf [file ac3c01747_si_001.pdf]

Supplementary Information:

Viscosity-sensitive membrane dyes as tools to estimate the crystalline structure of lipid bilayers

Miguel Paez-Perez[a], Michael R. Dent[a], Nicholas J. Brooks[a*] and Marina K. Kuimova[a*]

[a] *MSRH, Department of Chemistry, Imperial College London, Wood Lane, London, W12 0BZ, UK*
Corresponding author: m.kuimova@imperial.ac.uk

Experimental section:

- XRD analysis: Area per lipid (APL), tilt angle (θ), membrane thickness (d_{HH}) and Poisson ratio (ν) calculation.
- Custom processing of FLIM images

Supporting Figures:

Fig. S1: X-ray scattering intensity profiles for DOPC at 70% hydration.

Fig. S2: Effect of temperature on the membrane's structure.

Fig. S3: Example time-resolved BC10 traces and Laurdan emission spectra in DOPC and DPPC membranes

Fig. S4: Plot of membrane viscosity against Laurdan GP

Fig. S5: X-ray scattering intensity profiles for DPPC at 70% hydration.

Fig. S6: WAXS scattering intensity profiles at 70% hydration of POPC and DLPC.

Fig. S7: Membrane's viscosity-structure calibration in gel bilayers.

Fig. S8: Dependence of the parameter offset $\mathcal{P}_0$ on lipid composition.

Fig. S9: Time-resolved **BC10** fluorescence emission decay traces of *E. Coli* Polar Lipid Extract.

Fig. S9: WAXS trace of *E. Coli* Polar Lipid Extract.

Fig. S11: XRD characterization of OA effect on DOPC.

Fig. S12: FLIM characterization of DOPC:OA GUVs.

Fig. S13: XRD characterization of DOPC substitution by OA on DOPC:OA:DPPC:Chol membranes.

Fig. S14: Z-projections of **BC10**-stained DOPC:OA:DPPC:Chol GUVs.

Fig. S15: Close-up micrograph of 20:20:40:20 DOPC:OA:DPPC:Chol GUVs.

Fig. S16: Laurdan's GP in DOPC:OA:DPPC:Chol GUVs.

Supporting Tables:

Table S1: Fixed parameters for the estimation of lamellar properties using two SAXS peaks.

Table S2: Tabulated temperatures, amplitudes, lifetimes and viscosity obtained from BC10 in DOPC and DPPC membranes.

Table S3: Comparison of lifetimes and viscosities derived from the three membrane domains in Fig. S15.

Discussion:

- Interpretation of OA-induced changes in membrane composition.

Notes and references

Additional materials and methods:

Area per lipid (APL) and tilt angle (θ) calculation: APL was calculated from the WAXS traces, by fitting them to one or two Voigt functions together with a quadratic baseline function.

For the DPPC composition at $T<T_m$ (41°C), two peaks were required to fit the WAXS trace (20, at low q-values and 11, corresponding to the higher q-value). Then, the area per lipid chain was computed using the standard formula for an orthorhombic packing[1] as:

$$A_C = \frac{a_c\, b_c}{2} \qquad \text{(Eq. S5)}$$

where

$$a_c = 2d_{20} \quad b_c = \frac{d_{11}}{\sqrt{1-\left(\frac{d_{11}}{2d_{20}}\right)^2}} \qquad \text{(Eq. S6)}$$

And finally, lipid area was approximated as[2]:

$$A_L = \frac{2A_C}{\cos(\theta)} \qquad \text{(Eq. S7)}$$

With β being the lipid tilt angle, which itself was approximated from the bilayer thickness d_{HH} as[1]:

$$\cos(\theta) = \frac{d_{HH}}{2N(1.27\text{Å})+2D_H} \qquad \text{(Eq. S8)}$$

Where N is the number of carbons in the lipid tail (16 for DPPC) and D_H represents the headgroup thickness (assumed 3.1 Å).

On the other hand, the area per chain (A_C) and area per lipid (APL) for DOPC and DPPC (T>41°C) molecules approximated as suggested by *Mills et al.*[2] from the *q* position of the WAXS peak as:

$$\frac{APL}{2} \cong 1.32\left(\frac{9\pi}{4q}\right)^2 \qquad \text{(Eq. S9)}$$

When cholesterol was present, we considered the ideal case of lipid molecules located in a hexagonal lattice, so the addition of cholesterol would create a condensing effect on the lipid tails,[3,4] which is expected to decrease the APL of the phospholipid molecules. To account for this effect, we first computed the chain area as:[2]

$$A_{chain,total} = \frac{2}{\sqrt{3}}\left(\frac{2\pi}{q_0}\right)^2 \qquad \text{(Eq. S10)}$$

The phospholipid chain area was then calculated as:

$$A_{chain,PL} = \frac{A_{chain,total} - \chi_{chol}A_{chol}}{1-\chi_{chol}} \qquad \text{(Eq. S11)}$$

where A_{chol} is the estimated cross-sectional area of a cholesterol molecule (~30.6 Å^2, average from refs[5,6]). Once $A_{chain,PL}$ is known, we calculated the equivalent q_0 by solving Eq. S10, and then used that value to estimate the APL of the condensed phospholipid molecule according to Eq. S9.

Membrane thickness calculation: The diffraction profiles were obtained from the radial integration of the 2D SAXS and WAXS patterns, and peak position and width was determined by fitting them to pseudo-Voigt functions using a custom-built MATLAB® script. Membrane thickness was obtained from the two first diffraction peaks of the SAXS spectra using the method suggested by *Rappolt et al.*[7] as:

$$z_H = \pm\frac{d}{2\pi}\arccos\left(\frac{c1-\sqrt{8(r_F c_3)^2+8(c_2-r_F c_4)(r_F c_3)+c_1^2}}{4r_F c_3}\right) \qquad \text{(Eq. S12)}$$

with:

$$r_F = \frac{F1}{F2}; \qquad \text{(Eq. S13)}$$

$$c_1 = 2\sigma_H \exp\left[-2\left(\frac{\pi\sigma_H}{d}\right)^2\right];\quad c_2 = -|\rho_r|\sigma_C \exp\left[-2\left(\frac{\pi\sigma_C}{d}\right)^2\right];\qquad \text{(Eq. S14, S15)}$$

$$c_3 = 2\sigma_H \exp\left[-8\left(\frac{\pi\sigma_H}{d}\right)^2\right];\quad c_4 = -|\rho_r|\sigma_C \exp\left[-8\left(\frac{\pi\sigma_C}{d}\right)^2\right]\qquad \text{(Eq. S16, S17)}$$

And where: σ_H = Gaussian distribution for the headgroup, σ_C = Gaussian distribution for the tails and ρ_r = Minimum to maximum electron density ratio. Values for these parameters were taken from the literature and are shown below.

Table S1: Fixed parameters for the estimation of lamellar properties using two SAXS peaks.

Parameter	σ_H	σ_C	ρ_r
Value	3.1	4.4	0.9

Poisson ratio (ν) calculation: For lipid bilayers the Poisson ratio ν_{zx} relating the membrane's lateral (ε_A) and normal (ε_Z) deformations is described by:[8]

$$\nu_{zx} = -\frac{\varepsilon_A}{2\varepsilon_z}\qquad \text{(Eq. S18)}$$

where we defined the temperature-dependent in-plane area strain ε_A and the normal strain ε_Z as:

$$\varepsilon_A(T) = \frac{d_A(T)}{d_{A,T=5}} - 1\qquad \text{(Eq. S19)}$$

$$\varepsilon_A(T) = \frac{d_{HH}(T)}{d_{HH,T=5}} - 1\qquad \text{(Eq. S20)}$$

so that ν_{zx} is calculated from the slope in the ε_A vs ε_z plot.

Custom processing of FLIM images:
Because of the low **BC10** partitioning in the highly ordered membrane regions,[9,10] a low photon count number was obtained in these areas, and hence required a large binning in order to accumulate enough photon counts which would yield an accurate lifetime. However, such binning will include undesired regions (e.g. background and/or signal from adjacent domains), which could mask the distinct domains within a single GUV. To address this issue, we developed a custom-made analysis pipeline exploiting the model-free phasor-based lifetime analysis in addition to an adaptive binning strategy. Our approach can be summarized as follows:

1. **Adaptive binning:** Given a 2D array with the total number of photon counts per pixel, background pixels are suppressed by adaptive thresholding (column and row-wise calculation of the threshold value). Next, an additive kernel of increased size (S_k) is iteratively applied (i.e. static binning) so that:

$$P_{i,j} = \sum_{i'=i-S_k}^{i+S_k} \sum_{j'=j-S_k}^{j+S_k} P_{i',j'}\qquad \text{(Eq. S21)}$$

where $P_{i,j}$ represents the peak photon counts at in pixel (i,j). The optimal bin size was the minimal one which satisfied the constraint for a given number of peak photons counts per pixel. Hence, larger bins were applied in pixels of where the starting number of photons was lower. The adaptive binned image reconstruction was finally performed by combining the decay traces from the different optimal bin sizes.

2. **Image segmentation:** GUV detection was performed using the Circular Hough Transform. Fine-tuning of the region of interest (ROI) was then performed through radial integration of the detected circumference, which allowed to identify the GUV edge as the maximum of the radial profile. ROI shape could also be manually modified, if required.

3. **Phasor calculation:** The phasor (g_{ij},s_{ij}) coordinates were obtained for all the pixels above background level, after binning according to the expressions:

$$g_{i,j}(\omega) = \frac{\int_{t=0}^{t=T} I_{i,j}(t)\cos(\omega t)dt}{\int_{t=0}^{t=T} I_{i,j}(t)dt}\qquad \text{(Eq. S22)}$$

$$s_{i,j}(\omega) = \frac{\int_{t=0}^{t=T} I_{i,j}(t)\sin(\omega t)dt}{\int_{t=0}^{t=T} I_{i,j}(t)dt}\qquad \text{(Eq. S23)}$$

where $I_{i,j}(t)$ represents the photon counts at pixel (i,j) and time t; $\omega = 2\pi/f$ and $f = 1/T$ is the laser's repetition rate

4. **Phasor clustering and pixel classification:** A two-dimensional histogram of the phasor points belonging to each GUV was calculated, and local maxima ("hills") were used to identify the different clusters, subjected to a user-defined limit. In case the number of clusters was larger than a user-defined limit, the most spaced clusters were selected. Bins with a neglectable number of counts were considered noise and discarded, and the remaining phasor points were assigned to the closest cluster. This distribution was then used as the initial condition for building a two-dimensional Gaussian Mixture Model (GMM) based on the (g,s) pair distribution.
   GMM assumes all datapoints belong to a mixture of several Gaussian distributions (*e.g.* membrane domains, in our case) of unknown parameters. After finding these parameters, we computed the posterior probability of each phasor point to belong to a given cluster described by the Gaussian model. Importantly, this assigned a "soft" label to the different phasor points (i.e. the probability of belonging to a given cluster), as opposed to hard classification methods such as K-means (which will directly assign the query point to a given cluster). We exploited this fact to reject those points having ambiguous classification in further downstream processing. The rationale behind this choice was that, because of the binning strategy, pixels at the boundary between different regions could show an intermediate lifetime and phasor position. Therefore, by rejecting those, lifetime estimations would be more accurate.

5. **Lifetime calculation:** After phasor points were assigned to different clusters, a pool of decay traces was created for each ROI and cluster. These were then combined (which resulted in an increased SNR), and the resulting trace was used to calculate the fluorescence lifetime via LSQ fitting. Lifetimes were also calculated for whole clusters, and bootstrapping was used to quantify the error.

Supplementary figures:

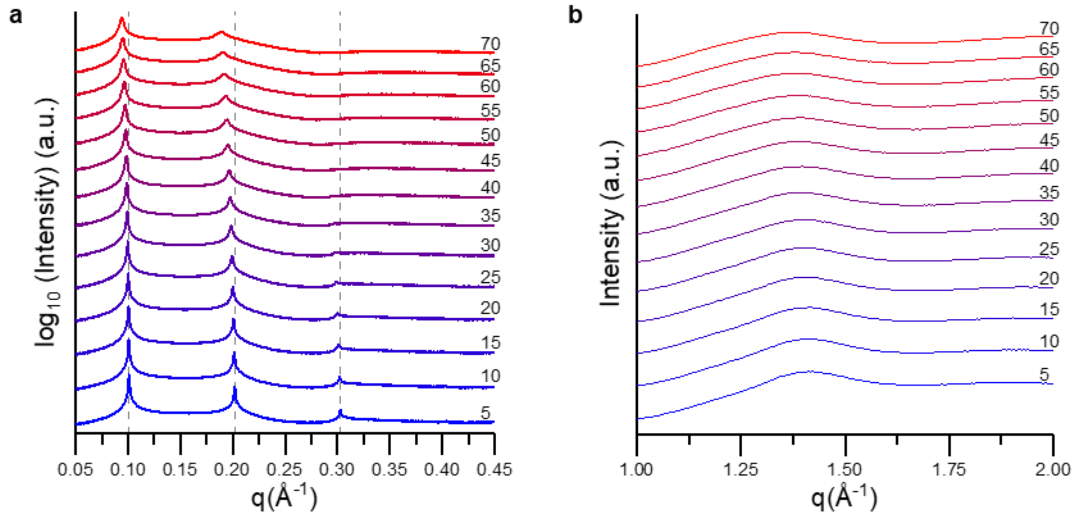

Fig. S1: X-ray scattering intensity profiles for DOPC at 70% hydration. (a) SAXS and (b) WAXS profiles at increasing temperature. The shift of WAXS peaks towards lower q-values is indicative of an increase in the APL.

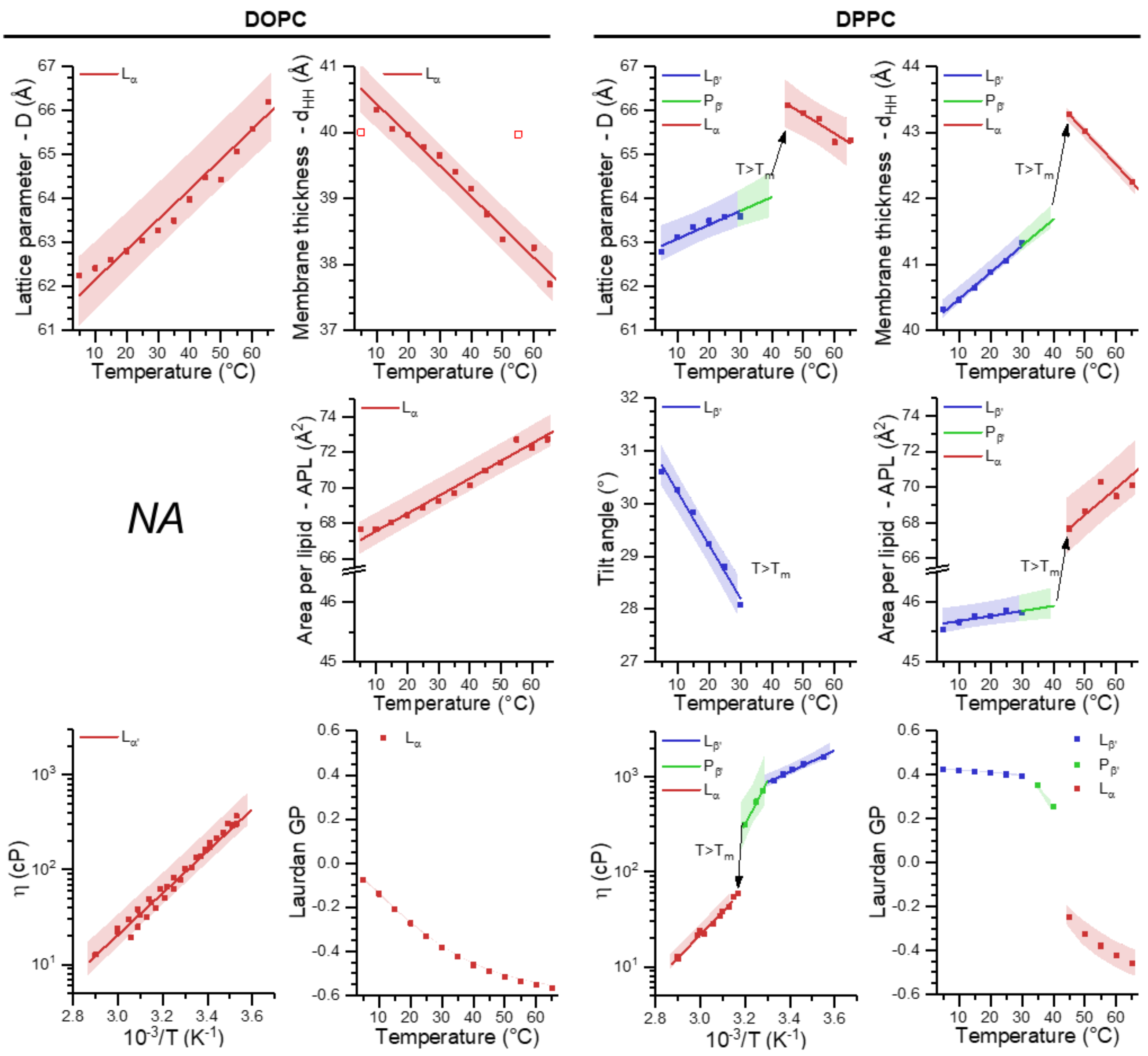

Fig. S2: Effect of temperature on the membrane's structure (lamellar distance D, membrane thickness d_{HH}, tilt angle θ and area per lipid APL), its viscosity η (evaluated by the lifetime of **BC10,** see Fig S3 for the raw data) and polarity (evaluated by Laurdan's GP, Fig S3). Note fluid membranes (*e.g.* DOPC and DPPC at T>41°C) lack a defined tilt angle.

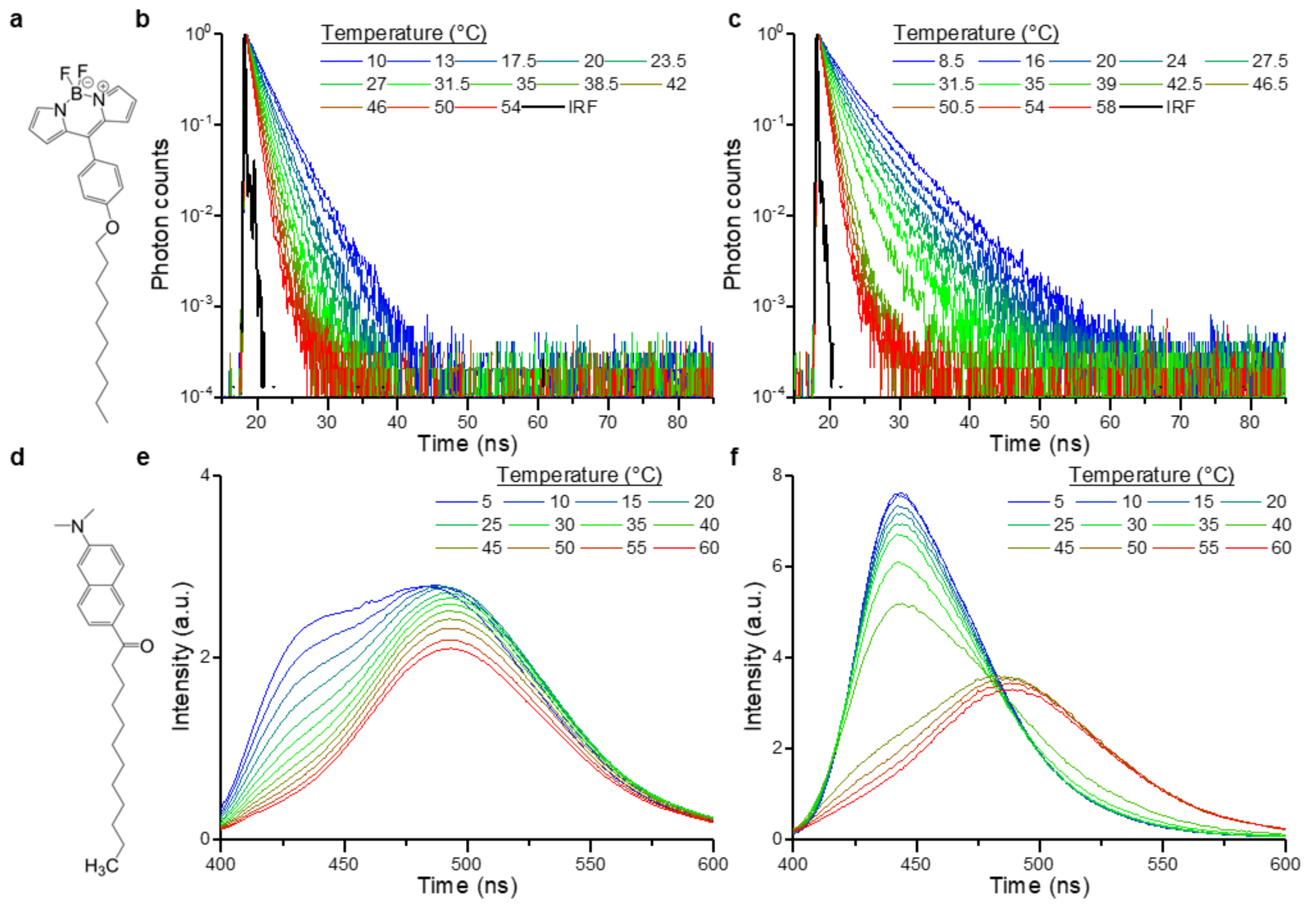

Fig. S3: Example time-resolved **BC10 (a)** decay traces for **b)** DOPC and **c)** DPPC LUVs; and Laurdan **(d)** emission spectra for **e)** DOPC and **f)** DPPC liposomes at increasing temperatures. While the time resolved decay traces seen for **BC10** in b) can be fitted by a monoexponential decay model (one dye environment), biexponential decay model is necessary in c) below 41C, consistent with the dye existing in two environments in the gel phase DPPC bilayers.[10]

Table S2: Tabulated temperatures, amplitudes, lifetimes and viscosity obtained from the BC10 time-resolved fluorescence decay traces in Fig. S3b,c. Note two decay components were used to reflect the two different localizations of BC10 in gel-phase membranes (T<41°C for DPPC bilayers), while a single exponential was used to fit the data obtained from lipid bilayers in the fluid phase.

Temperature	1/T	#Fit components	α1 (%)	α2 (%)	τ1 (ns)	τ2 (ns)	τ-Used(ns)	Viscosity (cP)
				**DPPC**				
**8.5**	0.0036	2	55%	45%	1.72	5.15	**5.15**	**1630.12**
**16**	0.0035	2	63%	37%	1.72	4.75	**4.75**	**1368.70**
**20**	0.0034	2	71%	29%	1.68	4.49	**4.49**	**1210.34**
**24**	0.0034	2	77%	23%	1.58	4.24	**4.24**	**1066.06**
**27.5**	0.0033	2	78%	22%	1.43	3.95	**3.95**	**914.37**
**31.5**	0.0033	2	80%	20%	1.28	3.56	**3.56**	**726.07**
**35**	0.0032	2	83%	17%	1.19	3.14	**3.14**	**551.35**
**39**	0.0032	2	83%	17%	1.02	2.42	**2.42**	**313.38**
**42.5**	0.0032	1	na	na	1.13	na	**1.13**	**59.05**

**43.85**	0.0032	1	na	na	1.09	na	**1.09**	**54.56**
**46.5**	0.0031	1	na	na	0.98	na	**0.98**	**43.15**
**49.5**	0.0031	1	na	na	0.93	na	**0.93**	**38.63**
**50.5**	0.0031	1	na	na	0.88	na	**0.88**	**34.22**
**54**	0.0031	1	na	na	0.80	na	**0.80**	**28.03**
**58**	0.0030	1	na	na	0.72	na	**0.72**	**22.21**
**60.5**	0.0030	1	na	na	0.75	na	**0.75**	**23.93**
**61**	0.0030	1	na	na	0.71	na	**0.71**	**21.55**
**72**	0.0029	1	na	na	0.56	na	**0.56**	**12.90**
**72**	0.0029	1	na	na	0.55	na	**0.55**	**12.16**
**DOPC**								
**10**	0.0035	1	na	na	2.61	na	**2.61**	**367.56**
**10**	0.0035	1	na	na	2.39	na	**2.39**	**302.79**
**10**	0.0035	1	na	na	2.39	na	**2.39**	**302.79**
**12**	0.0035	1	na	na	2.35	na	**2.35**	**293.69**
**13**	0.0035	1	na	na	2.40	na	**2.40**	**307.61**
**15**	0.0035	1	na	na	2.17	na	**2.17**	**246.68**
**17.5**	0.0034	1	na	na	2.04	na	**2.04**	**214.01**
**20**	0.0034	1	na	na	1.86	na	**1.86**	**175.19**
**20**	0.0034	1	na	na	1.94	na	**1.94**	**192.16**
**22**	0.0034	1	na	na	1.79	na	**1.79**	**161.56**
**23.5**	0.0034	1	na	na	1.67	na	**1.67**	**138.05**
**25**	0.0034	1	na	na	1.66	na	**1.66**	**136.27**
**27**	0.0033	1	na	na	1.47	na	**1.47**	**105.13**
**30**	0.0033	1	na	na	1.46	na	**1.46**	**103.76**
**31.5**	0.0033	1	na	na	1.28	na	**1.28**	**77.73**
**35**	0.0032	1	na	na	1.16	na	**1.16**	**62.78**
**35**	0.0032	1	na	na	1.32	na	**1.32**	**82.33**
**37**	0.0032	1	na	na	1.19	na	**1.19**	**66.11**
**38.5**	0.0032	1	na	na	1.05	na	**1.05**	**50.77**
**40**	0.0032	1	na	na	1.16	na	**1.16**	**61.99**
**42**	0.0032	1	na	na	0.94	na	**0.94**	**39.71**
**44**	0.0032	1	na	na	1.00	na	**1.00**	**45.38**
**45**	0.0031	1	na	na	1.03	na	**1.03**	**48.50**
**46**	0.0031	1	na	na	0.85	na	**0.85**	**31.26**
**49.65**	0.0031	1	na	na	0.88	na	**0.88**	**33.81**
**50**	0.0031	1	na	na	0.76	na	**0.76**	**25.07**
**50**	0.0031	1	na	na	0.92	na	**0.92**	**37.77**
**54**	0.0031	1	na	na	0.68	na	**0.68**	**19.19**
**55**	0.0030	1	na	na	0.83	na	**0.83**	**29.86**
**60**	0.0030	1	na	na	0.75	na	**0.75**	**24.05**

**60.65**	0.0030	1	na	na	0.72	na	**0.72**	**21.81**
**72.15**	0.0029	1	na	na	0.56	na	**0.56**	**12.75**

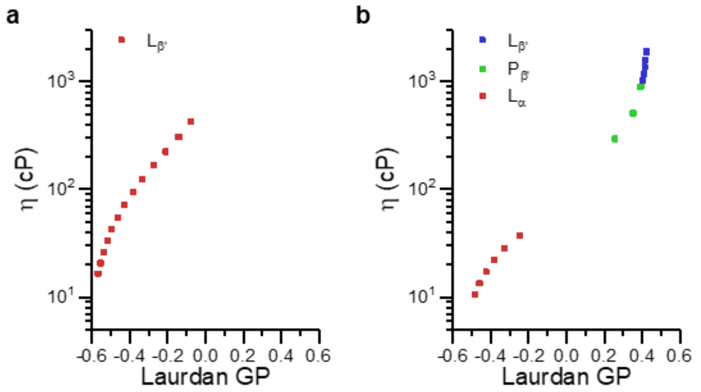

Fig. S4: Plot of membrane viscosity against Laurdan GP for **a)** DOPC and **b)** DPPC membranes

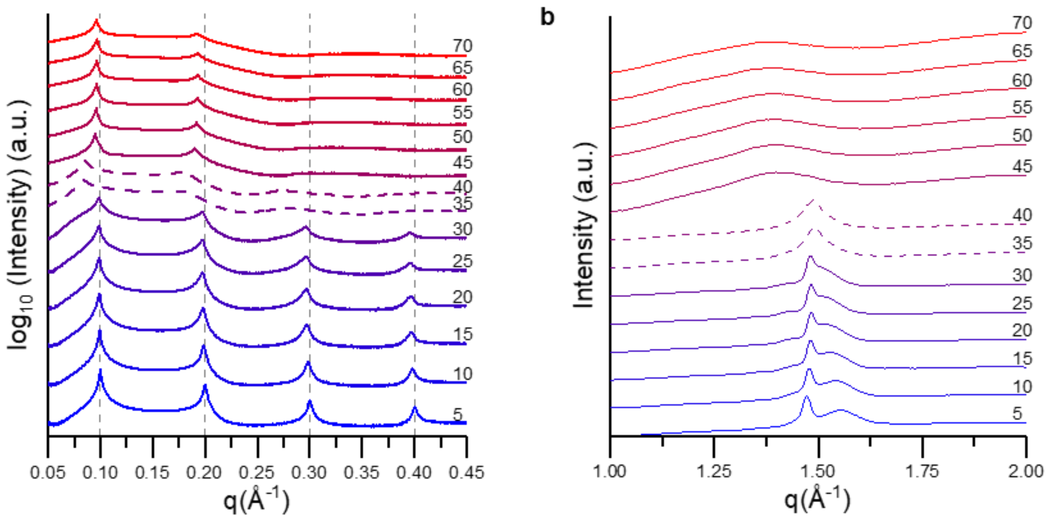

Fig. S5: X-ray scattering intensity profiles for DPPC at 70% hydration. **a)** SAXS and **b)** WAXS profiles at increasing temperature. Ripple phase is represented as a dashed line.

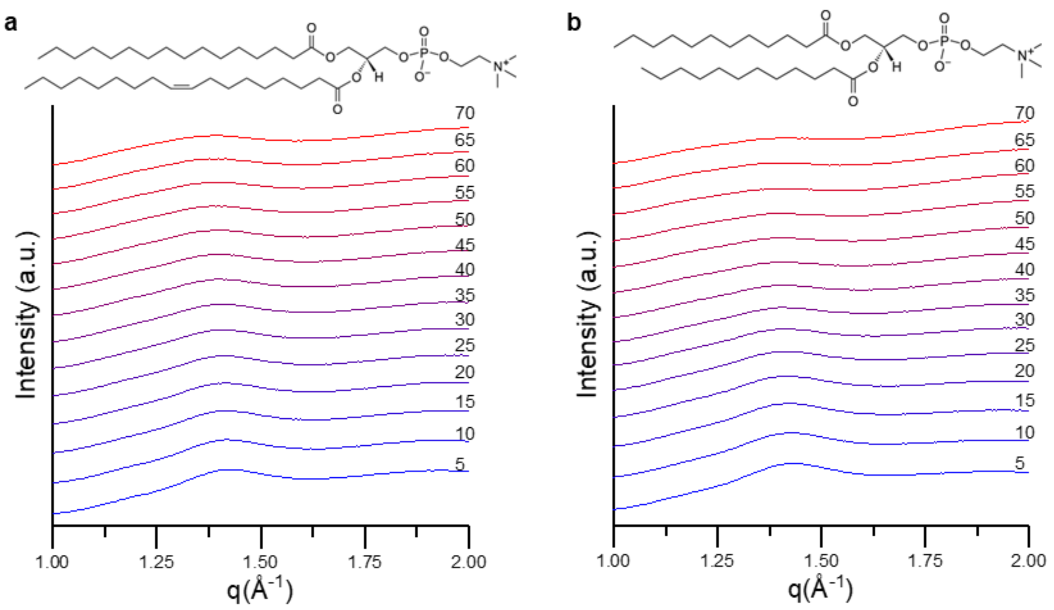

Fig. S6: WAXS scattering intensity profiles at 70% hydration of **a)** POPC and **b)** DLPC at increasing temperature.

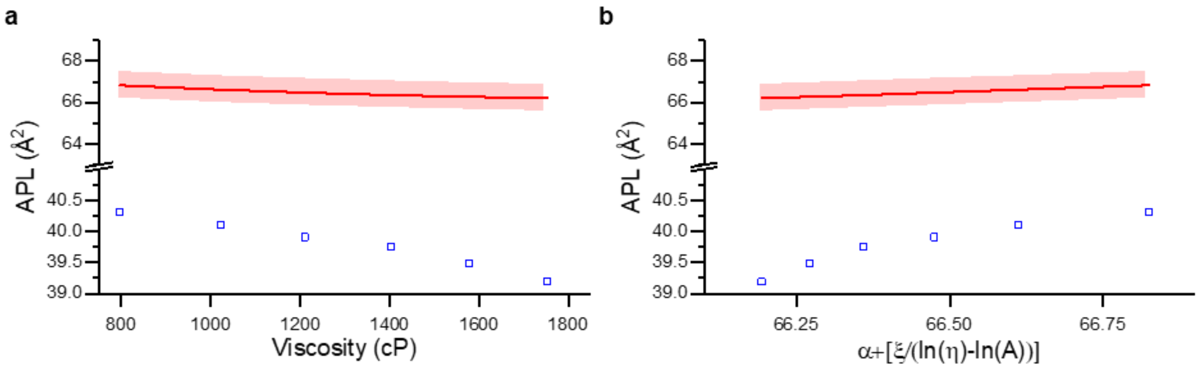

Fig. S7: Membrane's viscosity-structure calibration in fluid bilayers cannot be applied to gel bilayers. **a)** Relationship between microviscosity reported by **BC10**, calibrated using DOPC, and measured APL in DPPC. **b)** Relationship between APL and the transformed viscosity according to Eq. 8.

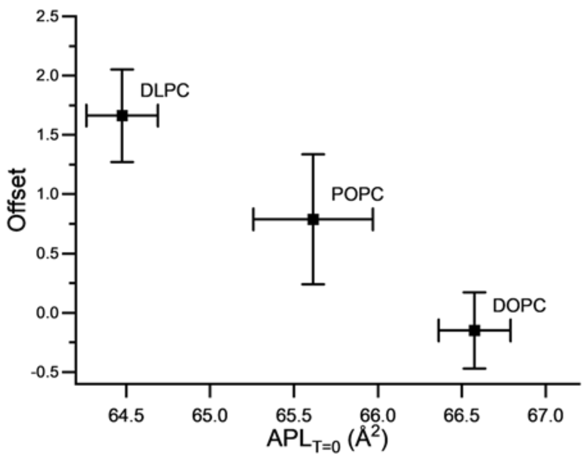

Fig. S8: Dependence of the parameter offset $\mathcal{P}_0$,used for calculating the structure-viscosity dependency of Fig. 3b, on the intrinsic characteristic of a different lipid molecules, here the APL at 0 K.

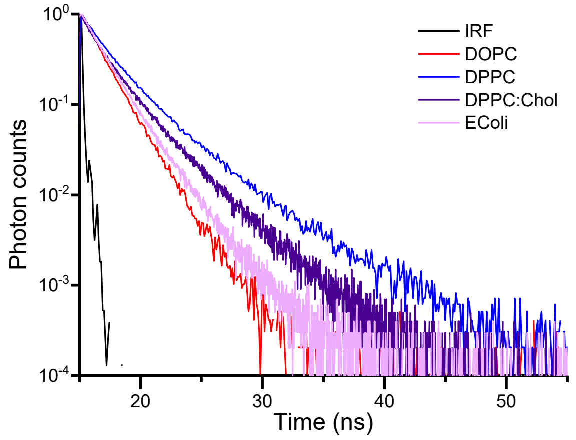

Fig. S9: Time-resolved **BC10** fluorescence emission decay traces of *E. Coli* Polar Lipid Extract (ECPLE) compared to model membranes, at 25C.

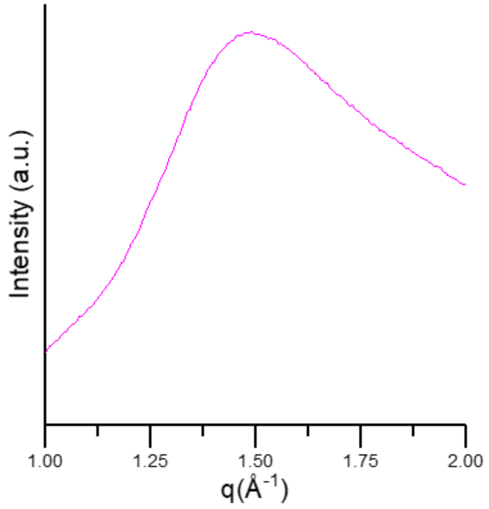

Fig. S9: WAXS trace of *E. Coli* Polar Lipid Extract (ECPLE) at 25C.

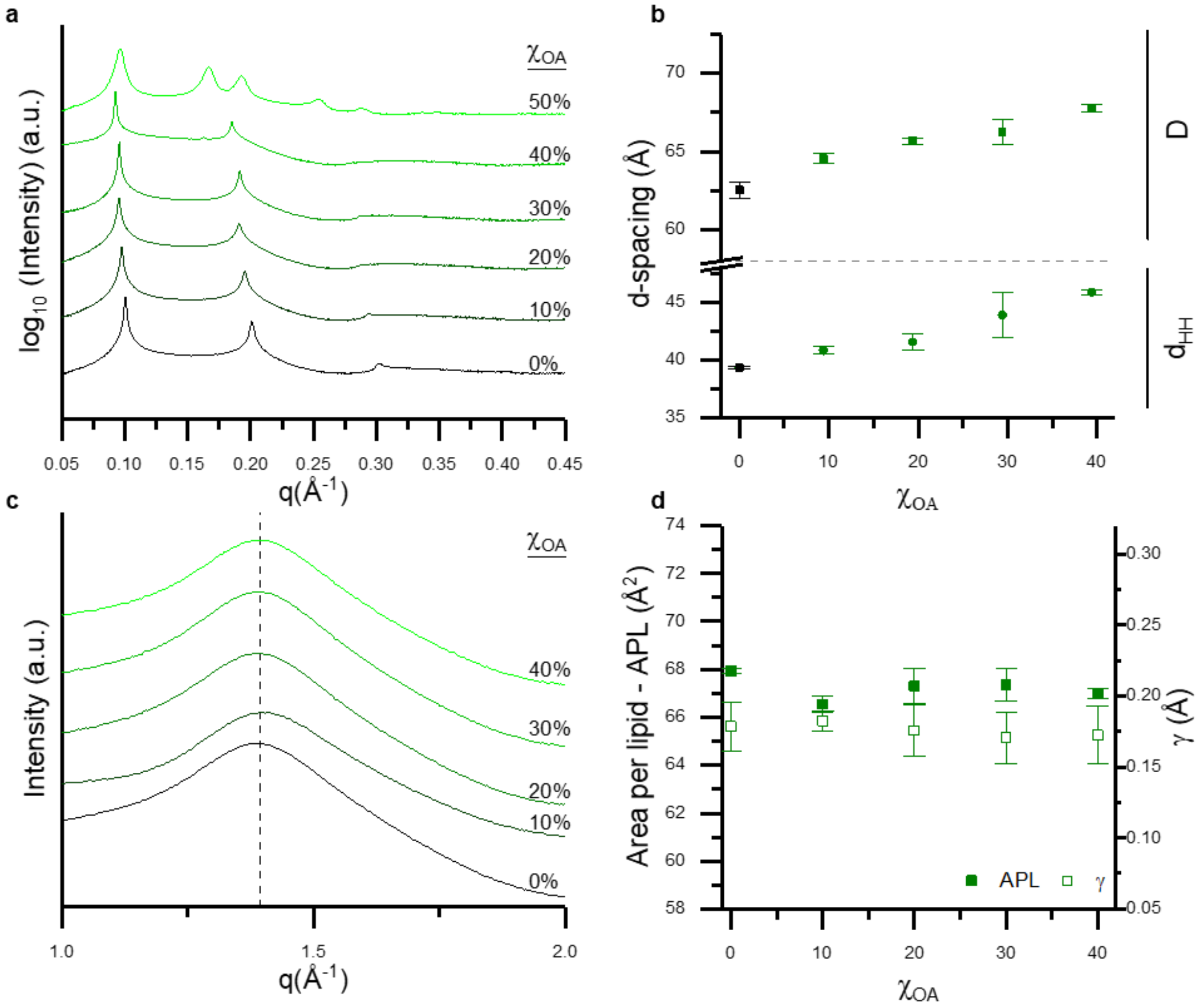

Fig. S11: XRD characterization of OA effect on DOPC. **a)** SAXS traces at increased %OA. At 50% OA the lipid architecture shifts to a H_{II} phase. **b)** Evolution of the lamellar repeat distance D and the headgroup-to-headgroup distance d_{HH} at increased %OA. **c)** WAXS traces showing the effect of OA on the membrane's lateral organization. **d)** Change in the APL and HWHM of the Lorentzian component of the fitted pseudo-Voigt.

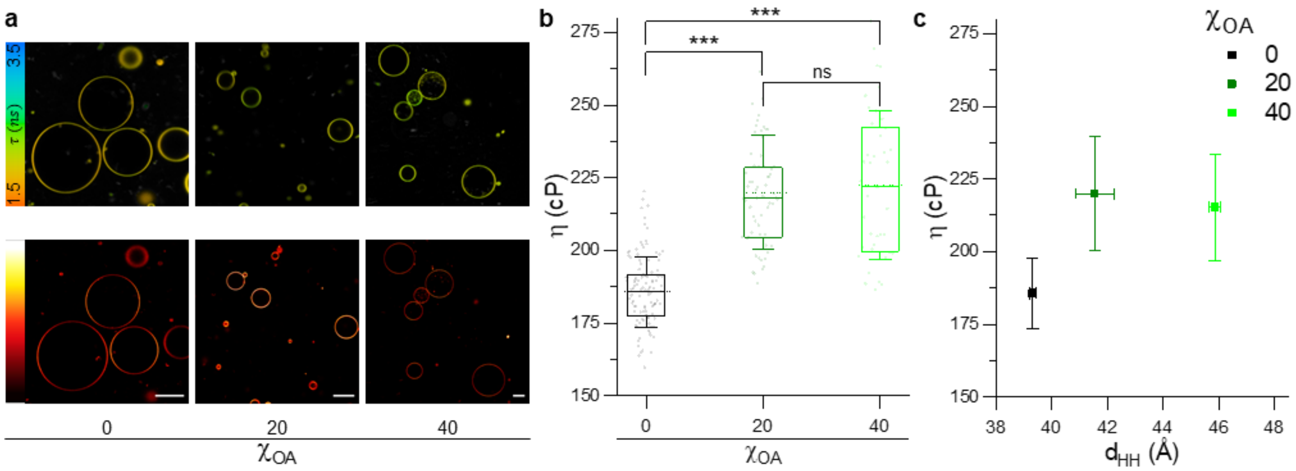

Fig. S12: FLIM characterization of DOPC:OA GUVs. **a)** Representative FLIM (top) and confocal (bottom) images showing **BC10** lifetime (top) and intensity (bottom) in DOPC:OA GUVs. Scale bar: 30 µm **b)** Box plot showing the change in viscosity upon OA addition. The calculated viscosities could be classified as a single group for each composition, suggesting no domains were present. **c)** Plot of membrane viscosity against membrane thickness at increasing OA concentration.

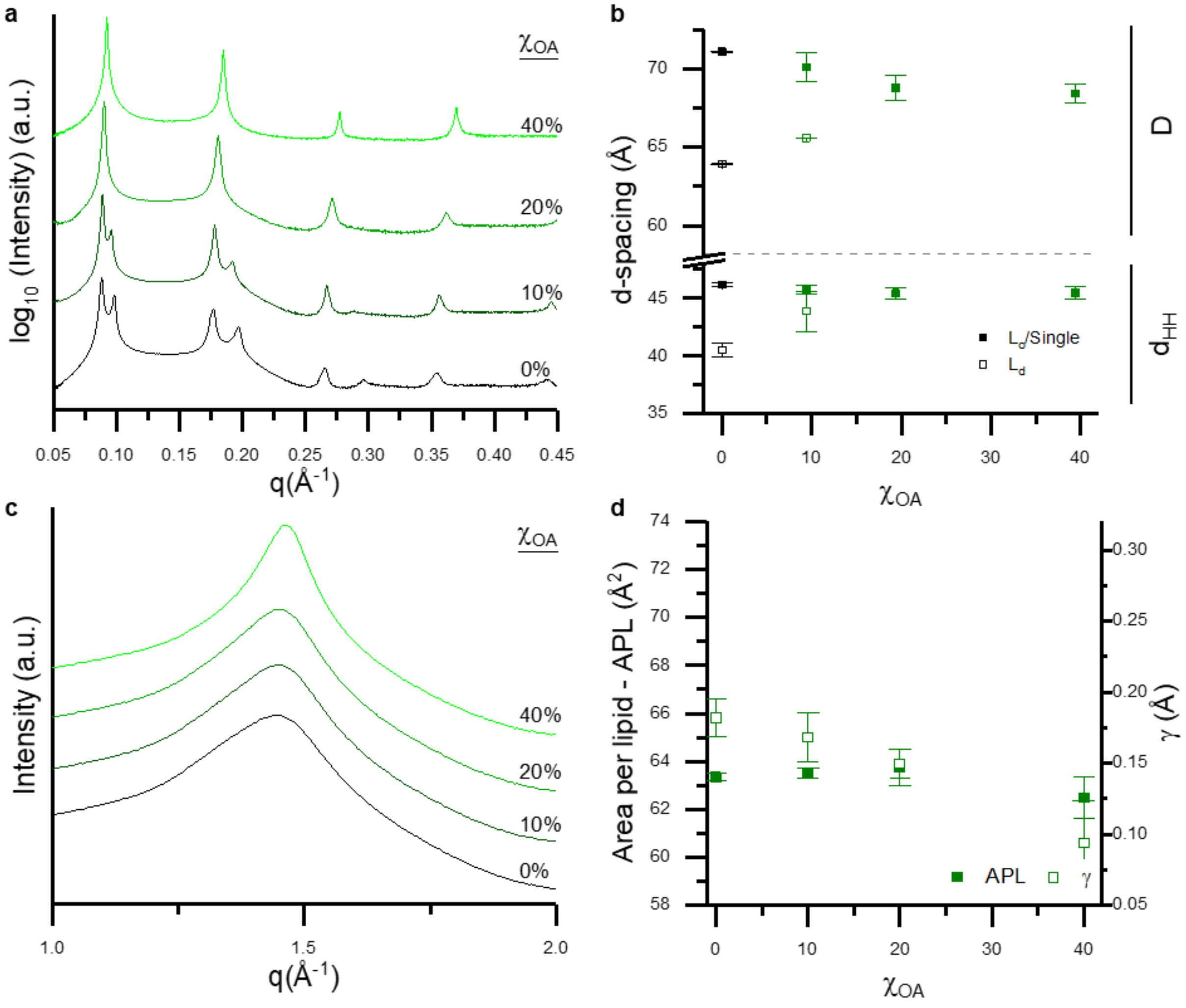

Fig. S13: XRD characterization of DOPC substitution by OA on DOPC:OA:DPPC:Chol membranes. **a)** SAXS traces at increased %OA. Above 20% OA, height mismatch between the two domains disappears. **b)** Evolution of the lamellar repeat distance D and the headgroup-to-headgroup distance d_{HH} at increased %OA. **c)** WAXS traces showing the effect of OA on the membrane's lateral organization. **d)** Change in the APL and HWHM of the Lorentzian component of the fitted pseudo-Voigt.

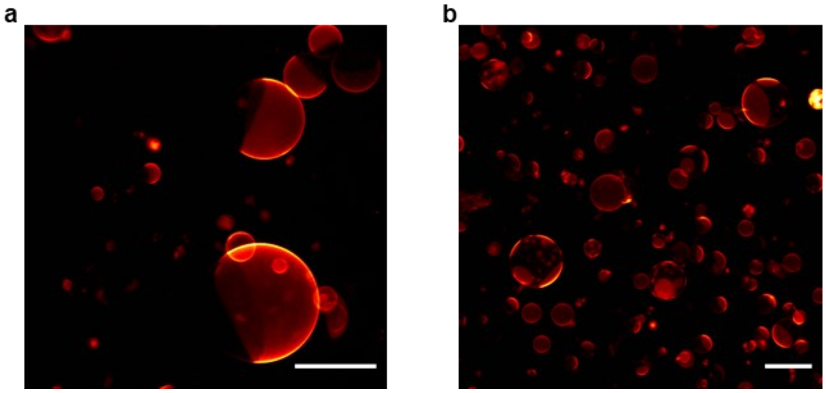

Fig. S14: Z-projections of **BC10**-stained DOPC:OA:DPPC:Chol GUVs. **a)** 40:0:40:20 and **b)** 20:20:40:20. The presence of smaller domains upon OA addition in b) is indicative of a lower line tension across the domain's boundary. The projections correspond to the confocal images shown in Fig. 3. Scalebar: 30 µm

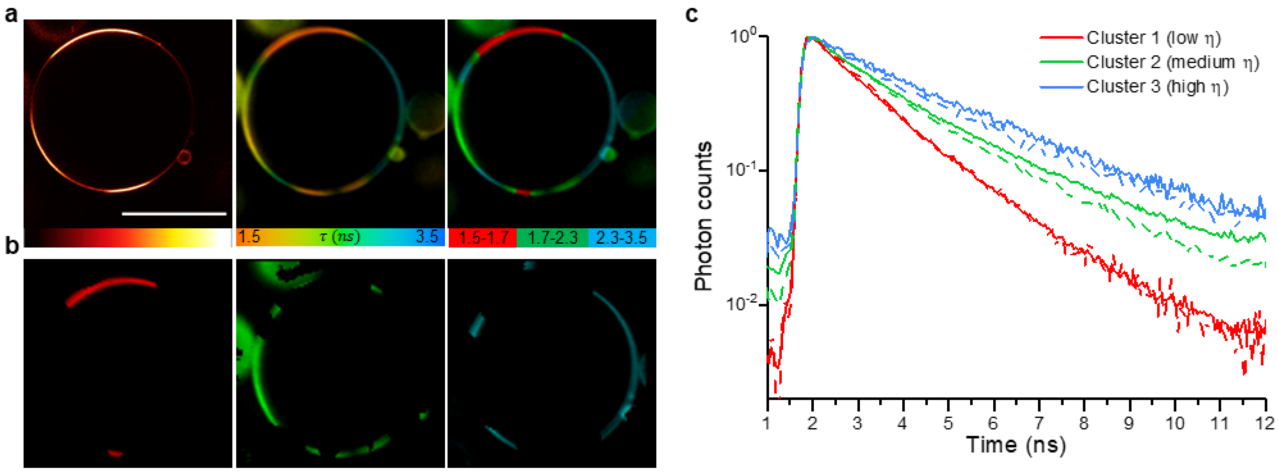

Fig. S15: Close-up micrograph of 20:20:40:20 DOPC:OA:DPPC:Chol GUVs showing coexistence of three regions of distinct viscosity. **a)** Confocal image (left) showing **BC10** intensity, continuous scale FLIM image (centre) and discrete-coloured FLIM image (right) showing **BC10** lifetime. **b)** Segmented image according to the measured lifetime. **c)** Fluorescence decay traces obtained by adding up all the individual pixel-wise decays in each of the identified clusters. Continuous lines represent the traces obtained using the custom adaptive-binning approach employed in the analysis, while dashed traces represent the decay traces obtained from binning in the commercially available SPCImage® software package. For high **BC10** intensity (Cluster1), which allows for a more accurate segmentation and requires a lower degree of binning, there is a full agreement between both approaches. The divergence observed in the intermediate cluster arises from variations in the masking and binning between the two tools, however, these are within experimental error (see Table S2).

Table S3: Comparison of lifetimes and viscosities derived from the three membrane domains in Fig. S15. The difference in viscosity (Δη) introduced by the analysis method is similar to the experimental one, therefore we deemed our approach was valid. Lifetime was obtained following monoexponential analysis of the binned decay traces

	$\tau_{classifier}$ (ns)	$\tau_{SPCImage}$ (ns)	$\eta_{classifier}$ (cP)	$\eta_{SPCImage}$ (cP)	Δη (method)	Δη (experimental)
Cluster 1	1.39	1.48	92.9	106.6	14	30
Cluster 2	1.88	2.1	179.8	228.9	49	45
Cluster 3	2.58	2.74	359.6	410.2	51	75

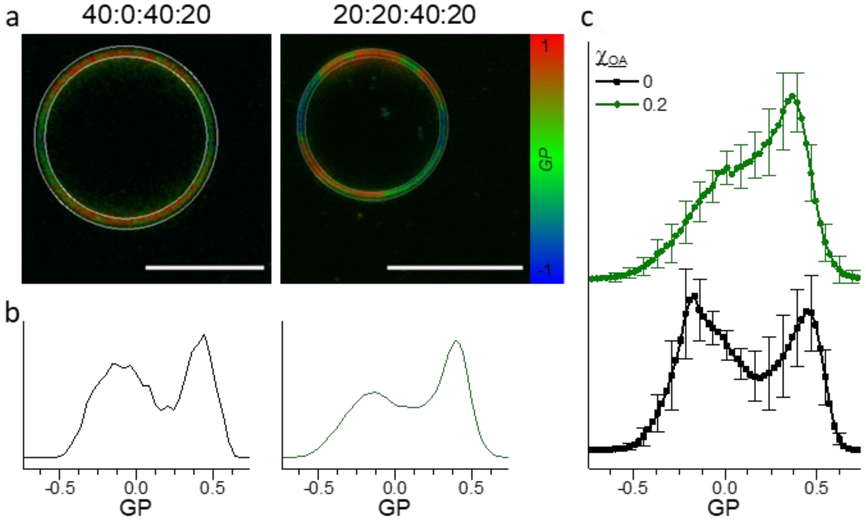

Fig. S16: Laurdan's GP in DOPC:OA:DPPC:Chol GUVs. **a)** Example of GP images of GUVs containing no (left) or 20% OA (right). Intensity contrast has been adjusted for visualization, and the region of interest (ROI) selected for the analysis is displayed as a white ring. Scalebar: 20 µm **b)** GP histogram for the GUVs shown in a). **c)** Histograms showing the GP distribution for all the analyzed GUVs (n>10). The presence of three domains in OA-containing GUVs can be inferred from the minimal number of Gaussian components needed to fit the GP histogram for the OA containing sample in c).

Interpretation of OA-induced changes in membrane composition:

Based on the combined SAXS/WAXS and FLIM characterization of OA-containing GUVs, we hypothesize the domain composition of OA-enriched GUVs could involve the following:

(i) *High viscosity* regions (η~465 cP): The membrane thickness and viscosity are similar to OA-free L_o domains, suggesting the relative DPPC:Chol ratio remains unchanged, and OA does not partition significantly into these regions. The estimated APL (51.9 $Å^2$) is also similar to that of the L_o region in OA-free GUVs.

(ii) *Low viscosity* regions (η~155 cP): These are likely domains which predominantly contain DOPC lipids.

(iii) *Intermediate viscosity* regions (η~250 cP): The viscosity values are similar to OA-saturated DOPC membranes (Fig. S12), while the membrane thickness is compatible to that of OA-free L_o domains, suggesting this region is rich in DOPC and OA. Remarkably, the ~0.9% decrease in the estimated APL with respect to the low viscosity regions is similar to previous OA-induced changes in the lipid area.[11]

Notes and references:

(1) Sun, W. J.; Tristram-Nagle, S.; Suter, R. M.; Nagle, J. F. Structure of Gel Phase Saturated Lecithin Bilayers: Temperature and Chain Length Dependence. *Biophys. J.* **1996**, *71* (2), 885–891.

(2) Mills, T. T.; Toombes, G. E. S.; Tristram-Nagle, S.; Smilgies, D. M.; Feigenson, G. W.; Nagle, J. F. Order Parameters and Areas in Fluid-Phase Oriented Lipid Membranes Using Wide Angle x-Ray Scattering. *Biophys. J.* **2008**, *95* (2), 669–681.

(3) Róg, T.; Pasenkiewicz-Gierula, M.; Vattulainen, I.; Karttunen, M. Ordering Effects of Cholesterol and Its Analogues. *Biochim. Biophys. Acta - Biomembr.* **2009**, *1788* (1), 97–121.

(4) Ma, Y.; Ghosh, S. K.; Dilena, D. A.; Bera, S.; Lurio, L. B.; Parikh, A. N.; Sinha, S. K. Cholesterol Partition and Condensing Effect in Phase-Separated Ternary Mixture Lipid Multilayers. *Biophys. J.* **2016**, *110* (6), 1355–1366.

(5) Chiu, S. W.; Jakobsson, E.; Jay Mashl, R.; Larry Scott, H. Cholesterol-Induced Modifications in Lipid Bilayers: A Simulation Study. *Biophys. J.* **2002**, *83* (4), 1842–1853.

(6) Hung, W.-C.; Lee, M.-T.; Chen, F.-Y.; Huang, H. W. The Condensing Effect of Cholesterol in Lipid Bilayers. *Biophys. J.* **2007**, *92* (11), 3960–3967.

(7) Rappolt, M. Bilayer Thickness Estimations with "Poor" Diffraction Data. *J. Appl. Phys.* **2010**, *107* (8), 084701.

(8) Terzi, M. M.; Deserno, M.; Nagle, J. F. Mechanical Properties of Lipid Bilayers: A Note on the Poisson Ratio. *Soft Matter* **2019**, *15* (44), 9085–9092.

(9) Wu, Y.; Štefl, M.; Olzyńska, A.; Hof, M.; Yahioglu, G.; Yip, P.; Casey, D. R.; Ces, O.; Humpolíčková, J.; Kuimova, M. K. Molecular Rheometry: Direct Determination of Viscosity in Lo and Ld Lipid Phases via Fluorescence Lifetime Imaging. *Phys. Chem. Chem. Phys.* **2013**, *15* (36), 14986.

(10) Dent, M. R.; López-Duarte, I.; Dickson, C. J.; Geoghegan, N. D.; Cooper, J. M.; Gould, I. R.; Krams, R.; Bull, J. A.; Brooks, N. J.; Kuimova, M. K. Imaging Phase Separation in Model Lipid Membranes through the Use of BODIPY Based Molecular Rotors. *Phys. Chem. Chem. Phys.* **2015**, *17* (28), 18393–18402.

(11) Leekumjorn, S.; Cho, H. J.; Wu, Y.; Wright, N. T.; Sum, A. K.; Chan, C. The Role of Fatty Acid Unsaturation in Minimizing Biophysical Changes on the Structure and Local Effects of Bilayer Membranes. *Biochim. Biophys. Acta - Biomembr.* **2009**, *1788* (7), 1508–1516.
